# Stereotactic ablative body radiotherapy for spinal metastasis from hepatocellular carcinoma: its oncologic outcomes and risk of vertebral compression fracture

**DOI:** 10.18632/oncotarget.20529

**Published:** 2017-08-24

**Authors:** Gyu Sang Yoo, Hee Chul Park, Jeong Il Yu, Do Hoon Lim, Won Kyung Cho, Eonju Lee, Sang Hoon Jung, Youngyih Han, Eun-Sang Kim, Sun-Ho Lee, Whan Eoh, Se-Jun Park, Sung-Soo Chung, Chong-Suh Lee, Joon Hyuk Lee

**Affiliations:** ^1^ Department of Radiation Oncology, Samsung Medical Center, Sungkyunkwan University School of Medicine, Seoul, Korea; ^2^ Department of Neurosurgery, Samsung Medical Center, Sungkyunkwan University School of Medicine, Seoul, Korea; ^3^ Department of Orthopedic Surgery, Samsung Medical Center, Sungkyunkwan University School of Medicine, Seoul, Korea; ^4^ Department of Medicine (Division of Hepatology), Samsung Medical Center, Sungkyunkwan University School of Medicine, Seoul, Korea; ^5^ Department of Medical Device Management and Research, Samsung Advanced Institute for Health Sciences and Technology, Sungkyunkwan University, Seoul, Korea; ^6^ Department of Radiation Oncology, Samsung Changwon Hospital, Sungkyunkwan University School of Medicine, Changwon-si, Korea

**Keywords:** stereotactic ablative body radiotherapy, spine, hepatocellular carcinoma, vertebral compression fracture

## Abstract

Spinal metastases from hepatocellular carcinoma (HCC) require high-dose irradiation for durable pain and tumor control. Stereotactic ablative body radiotherapy (SABR) enables the delivery of high-dose radiation. However, but vertebral compression fracture (VCF) can be problematic. The aim of his study is to evaluate the outcome and risk of VCF after SABR for spinal metastasis from HCC. We retrospectively reviewed 33 lesions in 42 spinal segments from 29 patients who received SABR with 1 fraction (16-20 Gy), or 3 fractions (18-45 Gy) from September 2009 to January 2015. The 1-year local control (LC) rate was 68.3%. Radiographic grade of cord compression (RGCC) was the only independent prognostic factor associated with LC (*P* = 0.007). The 1-year ultimate LC rate including the outcome of salvage re-irradiation was 87.2%. The pain response rate was 73.3% according to the categories of the International Bone Metastases Consensus Group. The 1-year VCF-free rate was 71.5%. Pre-existing VCF (*P* < 0.001) and only-lytic change (*P* = 0.017) were associated with a higher post-SABR VCF rate. One-third of post-SABR VCFs required interventions. SABR for spinal metastases from HCC provided efficacious LC, especially for lesions with RGCC ≤ II, and showed effective and durable pain relief. As VCF after SABR occurred frequently for vertebral segments with pre-existing VCF and only-lytic change, early preventive vertebroplasty is considerable for those high-risk vertebral segments.

## INTRODUCTION

Hepatocellular carcinoma (HCC) is the fifth most common cancer worldwide and the second leading cause of cancer-related death [1]. Although distant metastasis is less likely in HCC than other tumors, the incidences have increased in the last decade as the overall survival (OS) of patients with metastatic HCC has improved [2, 3]. Therefore, efficient palliation of metastasis from HCC has become an important clinical issue.

Spinal metastasis is estimated to represent 40% of bone metastases from HCC [4]. Radiotherapy (RT) is the most commonly used modality for spinal metastasis from HCC. RT of 30 Gy in 10 fractions is widely accepted as conventional regimen for spinal metastasis. However, as dose-response relationships between RT dose and symptom palliation have been reported, and conventional RT for spinal metastasis from HCC showed a high retreatment rate of up to 50%, there have been efforts to deliver a higher dose to spinal metastases from HCC [4–6].

Stereotactic ablative body radiotherapy (SABR) enables delivery of high-dose radiation to a specific target. Due to recent technological advances, SABR is now considered an effective modality for spinal metastasis. However, SABR can result in some orthopedic complications, such as vertebral compression fracture (VCF) or neuropathies [7]. Although there are a limited number of studies showing the effectiveness of SABR for spinal metastasis from HCC in local control (LC) and pain palliation [8, 9], there have not been any reports evaluating the risk of VCF in company with the oncologic outcome.

In this study, we aimed to evaluate the oncologic outcome and risk of VCF after SABR for spinal metastasis from HCC. We also identified the prognostic factors related to oncologic outcome and post-SABR VCF.

## RESULTS

### Patient and tumor characteristics

The patient and tumor characteristics are summarized in Table 1. Twenty-five (86.2%) patients were Child-Pugh classification A. The Cancer of the Liver Italian Program (CLIP) score was 2 or less for 22 patents (75.9%). Ten patients (34.5%) had visceral metastasis additionally. One patient received fixation before SABR. More than half (54.5%) of the lesions showed radiographic grade of cord compression (RGCC) ≤ II. About 64% of the lesions showed no neurologic symptom (neurological grade a) before SABR. The median age was 55 years (range, 35-74 years). The median tumor volume was 21.3 cc (range, 2.6-221.27 cc). The median follow-up was 7 months (range, 1-43 months).

**Table 1:** Patients and tumor characteristics (no. of patients = 29, no. of lesions = 33)

Characteristics	Values (%)
Sex	
Male	28 patients (96.5)
Female	1 patient (3.5)
Child Pugh class	
A	25 patients (86.2)
B	4 patients (13.8)
CLIP score	
0	7 patients (24.1)
1	5 patients (17.2)
2	10 patients (34.5)
3	5 patients (17.2)
4	1 patient (3.5)
5	1 patient (3.5)
ECOG performance scale*	
0-1	22 patients (88.0)
2-4	3 patients (12.0)
Solitary bone metasiasis	
Yes	14 patients (48.3)
No	15 patients (51.7)
Other metastasis	
Extraspinal bone metastasis	11 patients (37.9)
Visceral metastasis	10 patients (34.5)
Tumor location	
C spine	5 lesions (15.2)
C and T spine	0 lesion (0.0)
T spine	13 lesions (39.4)
T and L spine	4 lesions (12.1)
L spine	10 lesions (30.3)
L spine and sacrum	1 lesion (3.0)
Previous treatment to lesion of interest	
Radiotherapy	2 lesions (6.1)
Fixation	1 lesion (3.0)
Radiation dose	
16 Gy/1fx	3 lesions (9.1)
18 Gy/1fx	18 lesions (54.6)
20 Gy/1fx	9 lesions (27.3)
18 Gy/3fx	1 lesion (3.0)
36 Gy/3fx	1 lesion (3.0)
45 Gy/3fx	1 lesion (3.0)
Radiographic grade of cord compression	
0 Spine bone involved only	7 lesions (21.2)
I Thecal sac impinged	7 lesions (21.2)
II Thecal sac compressed	4 lesions (12.1)
III Spincal cord impinged	10 lesions (30.3)
IV Cord displaced, CSF visible between cord and tumor	5 lesions (15.2)
Neurological grade of cord compression^†^	
a No neurological abnormality	18 lesions (64.3)
b Focal minor symptom (e.g., radiculopathy, sensory change)	7 lesions (25.0)
c Functional paresis (≥ 4/5 muscle power)	3 lesions (10.7)

CLIP, Cancer of the Liver Italian Program; ECOG, Eastern Cooperative Oncology Group (ECOG); C, Cervical; T, Thoracic; L Lumbar; fx, Fraction; CSF, Cerebrospinal Fluid.

*Information for ECOG was available only for 25 patients.

^†^Information for neurological grade of cord compression was available only for 28 lesions.

### Oncologic outcomes

**Figure 1:**
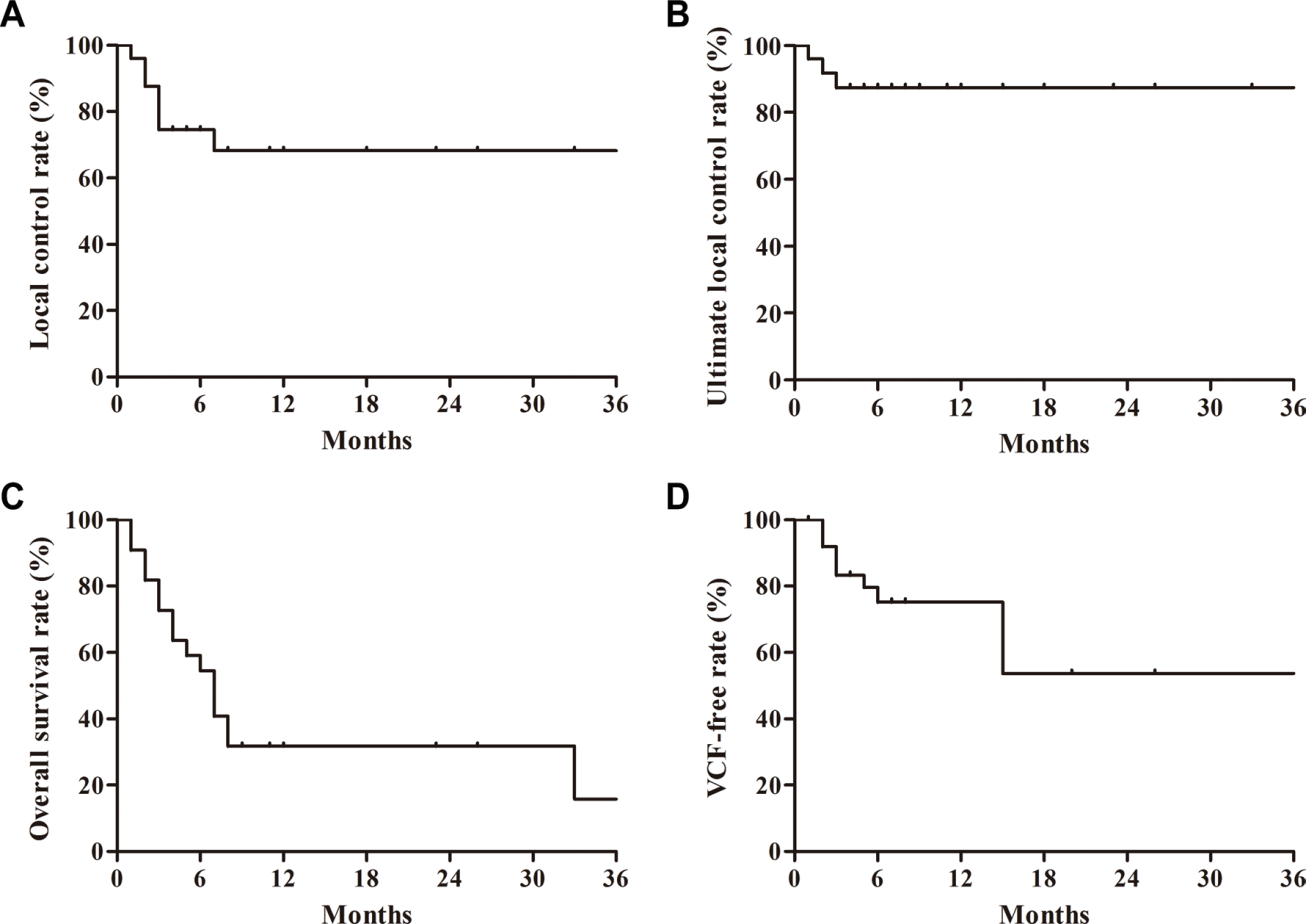
**(A)** Actuarial local control rate, **(B)** actuarial ultimate local control rate, **(C)** actuarial overall survival rate, and **(D)** actuarial vertebral compression fracture (VCF) free rate after stereotactic ablative body radiotherapy.

Twenty-five of 33 lesions were evaluable by follow-up images. Partial remission and stable disease status were achieved for 2 and 16 lesions, respectively. The 6-month and 1-year LC rates were 74.5% and 68.3%, respectively (Figure 1A). Seven of 25 lesions showed local failure during the follow-up. Six of the 7 progressed lesions showed epidural progression near the spinal cord, and 1 lesion within the vertebral body.

RGCC ≤ II was the only independent prognostic factor related to better LC on multivariate analyses (*P* = 0.007; Table 2). Minimal dose (D_min_) at gross tumor volume (GTV) < 35 Gy in biologically effective dose with α/β of 10 (Gy_10_) was associated with worse LC with marginal significance in univariate analysis (*P* = 0.053). However, there was significant correlation between RGCC and D_min_ at GTV (*P* = 0.014), thereby, this variable was excluded from multivariate analysis. Four of 7 progressed lesions were re-irradiated with 30–50 Gy in 10 fractions. Salvage RT was done with intensity-modulation technique to minimize the spinal cord dose. Three of them achieved ultimate LC during the follow-up. The 1-year ultimate LC rates including the outcome of salvage re-irradiation were 87.2% (Figure 1B).

**Table 2:** Univariate and multivariate analyses for local control and overall survival

Factors	Univariate analysis				Multivariate analysis	
	LC		OS		LC	OS
	At 6 months (%)	*P*-value	At 6 months (%)	*P*-value	*P*-value	*P*-value
Child Pugh classification		0.387		0.011	NA	NA
A	72.1		64.3			
B	100.0		25.0			
CLIP score		0.717		0.001	0.260	0.029
0-2	76.2		73.7			
2-5	50.0		14.3			
ECOG performance scale		0.372		0.008	NA	0.136
0-1	72.0		64.2			
2-4	100.0		25.0			
Solitary bone metastasis*		0.973		0.145	NA	NA
No	65.6		46.7			
Yes	64.8		70.1			
Visceral metastasis		0.302		0.052	0.225	0.083
No	69.7		67.1			
Yes	85.7		38.6			
BM outside spine		0.387		0.020	0.617	0.321
No	69.3		71.1			
Yes	85.7		34.1			
RGCC		0.001		0.395	0.007	0.885
0-II	92.9		68.8			
III-V	45.0		40.9			
Prescribed dose		0.981		0.195	0.257	0.812
< 60 Gy_10_	71.8		47.7			
≥ 60 Gy_10_	78.8		72.7			
D_min_ at GTV		0.053		0.360	NA	NA
< 35 Gy_10_	61.9		44.8			
≥ 35 Gy_10_	90.0		80.0			

LC, Local Control; OS, Overall Survival; NA, Not Analyzed; CLIP, Cancer of the Liver Italian Program; ECOG, Eastern Cooperative Oncology Group; BM, Bone Metastasis; RGCC, Radiographic Grade of Cord Compression; Gy_10_, Gy of Biologically Effective Dose with α/β=10; D_min_, Minimal Dose; GTV, Gross Tumor Volume.

*Solitary bone metastasis was excluded in multivariate analysis because of its significant correlations with visceral metastasis (*P* < 0.001) and BM outside spine (*P* < 0.001).

The 1-year OS rate was 38.9% (Figure 1C). The median survival was 7 months (95% confidence interval, 5.03-8.97 months). On multivariate analysis, only CLIP score was identified as an independent prognostic factor related to OS (*P* = 0.029; Table 2). Child-Pugh classification, which was associated with OS on univariate analysis (*P* = 0.011; Table 2), was excluded from the multivariate analysis because of significant correlation with CLIP score (*P* = 0.001).

### Post-SABR VCF and other toxicities

Forty-two segments were evaluable for post-SABR VCF. We observed 6 *de novo* VCFs and 6 progressions of pre-existing VCFs during the follow-up. Baseline spinal instability neoplastic score (SINS) components and final classification of each treated segment according to post-SABR VCF status are summarized in Table 3. The 1-year and 2-year VCF-free rates were 71.6% and 51.2%, respectively (Figure 1D). The 1-year and 2-year *de novo* VCF-free rates were 85.4% and 61%, respectively. On multivariate analysis, only pre-existing VCF was an independent prognostic factor related to VCF after SABR (*P* = 0.03; Table 4). However, we observed no post-SABR VCF in patients with mixed type of lesion: therefore, lesion type, which was associated with post-VCF on univariate analysis, was excluded in the multivariate analysis. Among the segments showing post-SABR VCF, vertebroplasty was applied to 3 lesions, and fixation with screw was used for 1 lesion.

**Table 3:** Scores according to SINS component and final classification of each treated vertebral segment

Factor	Post-SABR VCF (N = 12 segments)	No Post-SABR VCF (N = 30 segments)	% of VCF
Location			
Junctional	5	15	25.0
Mobile	4	8	33.3
Semi-rigid	3	7	30.0
Rigid	0	0	-
Pain			
Mechanical	8	20	28.6
Occasional and non-mechanical	1	5	16.7
None	3	5	37.5
Bone lesion type			
Only-lytic	12	20	37.5
Mixed (lytic and blastic)	0	10	0.0
Alignment			
Subluxation/translation	1	0	100.0
Kyphosis/scoliosis	0	0	-
Normal	11	30	36.7
Vertebral body collapse			
≥ 50%	2	0	100.0
< 50%	4	4	100.0
No collapse by > 50% of the bodies affected by tumor	1	2	33.3
None of the above	5	24	17.2
Posterior element involvement			
Bilateral	1	1	50.0
Unilateral	0	5	0.0
Not involved	11	24	31.4
SINS			
Stable (0-6)	3	16	15.8
Indeterminate instability (7-12)	8	14	36.4
Unstable (13-)	1	0	100.0

SABR, Stereotactic Ablative Body Radiotherapy; VCF, Vertebral Compression Fracture; SINS, Spinal Instability Neoplastic Score.

**Table 4:** Univariate and multivariate analyses for vertebral compression fracture-free survival

Variable	Univariate analysis		Multivariate analysis	
	1yr-VCFFR (%)	*P*-value	HR (95% CI)	*P*-value
Baseline SINS				
≤ 6	87.4	0.037	0.453 (0.074-2.765)	0.391
> 6	59.8			
Lesion type				
Mixed type	100.0	0.017	NA	NA
Lytic only	61.8			
Previous VCF before SABR				
No	85.4	< 0.001	0.199 (0.046-0.859)	0.030
Yes	22.5			
Maximum dose on segment				
< 20 Gy	74.5	0.689	0.814 (0.236-2.803)	0.744
≥ 20 Gy	70.4			
Pain				
Mechanical	72.0	0.872	1.171 (0.319-4.301)	0.813
Non-mechanical or none	68.1			

VCFFR, Vertebral Compression Fracture-Free Rate; HR, Hazard Ratio; 95% CI, 95% Confidence Interval; SINS, Spinal Instability Neoplastic Score; NA, Not Analyzed; VCF, Vertebral Compression Fracture; SABR, Stereotactic Ablative Body Radiotherapy.

No patient represented radiation-induced neuropathy, even for patients who received re-irradiation during the follow-up. There was no neuropathy associated with post-SABR VCF, either. One patient, who received SABR of 20 Gy in single fraction to cervical spines, experienced headache of grade 2. One patient who received SABR of 18 Gy in a single fraction to thoracic spine experienced dysphagia of grade 1.

### Pain and neurologic symptom responses

The pre-SABR pain statuses were evaluable only in 23 of 33 lesions. Twenty-one of 23 lesions led to pain with numeric rating system (NRS) from 2 to 10, and 2 lesions showed no pain. Only Fifteen (45.5%) of 33 lesions were available in analysis of pain response. The median NRS score decreased from 6 (range, 0-10) to 2 (range, 0-7) after SABR. The difference of median NRS before and after SABR was significant (*P* = 0.001). The median oral morphine equivalent dose (OMED) also decreased from 23 mg (range, 0-155) to 15 mg (range, 0-150) after SABR, however, the difference was not statistically significant (*P* = 0.564). Crude pain response rate according to the International Bone Metastases Consensus Group (IBMCG) was 73.3%. Complete response rate and partial response rate were 33.3% and 40%, respectively. Pain palliation occurred at median of 1 week (range, 1-5 weeks) after SABR. Median pain control duration of pain responders was 7 months (range, 2-8 months). One lesion showed stable pain response. This lesion presented no pain before SABR. Therefore, no analgesic was administrated during the follow-up. Pain progression after SABR occurred in 1 lesion which accompanied progression of pre-existing VCF. Other 2 lesions showed indeterminate responses with increased OMED and decreased NRS score.

The neurologic symptom statuses before and after SABR were evaluable in 28 of 33 lesions. Among 28 lesions, 18 lesions represented neurological grade a before SABR. After SABR for these 18 lesions, the neurological grades were all stable. Among the 10 lesions representing neurologic symptoms before SABR, 7 lesions showed the neurological grade b, and 3 lesions did the grade c before SABR. Among those 7 lesions with neurological grade b, neurologic symptoms disappeared in 6 lesions (85.7%) after SABR. However, 1 lesion represented persistent symptom of neurological grade b after SABR. The median time interval between the onset of neurologic symptom and SABR of those lesions with grade b was 30 days (range, 6-90 days). The time interval between the onset of neurologic symptom and SABR of the lesion with persistent neurological symptom after SABR was 21 days. Among those 3 lesions with neurological grade c, neurologic symptoms disappeared in 2 lesions (66.7%). The median time interval between onset of symptom and SABR of those 3 lesions was 11 days (range, 10-13 days). The motor weakness was persistent in 1 lesion of which the time interval between onset of symptom and SABR was 11 days. There is no significant correlation between time interval from onset of neurologic symptom to SABR and the neurologic response.

## DISCUSSION

In the present study, we evaluated the oncologic outcome and the risk of post-SABR VCF for spinal metastasis from HCC which requires a high-dose irradiation, possibly by SABR, and at the same time, is vulnerable to post-SABR VCF.

In the present study, 1-year LC rate of all lesions was 68.3%. For lesions with RGCC ≤ II, 1-year LC rate was 92.9% which is comparable to previous results of other literatures reporting LC as 80-90% [10]. The median D_min_ at GTV for lesions with RGCC ≤ II was 37.1 Gy_10_ (range, 15.6-96.0 Gy_10_). For lesions with RGCC ≥ III, however, 1-year LC rate was 0%, and the median D_min_ at GTV in this group was only 24.8 Gy_10_ (range, 9.1-41.9 Gy_10_). This difference in D_min_ at GTV between the 2 groups (RGCC ≤ II vs. ≥ III) resulted from the inevitable dose reduction at the area of tumor contacting with the neural structure for spinal cord sparing in the group of RGCC ≥ III. Nevertheless, for this group, the 1-year ultimate LC rate including the outcome of salvage re-irradiation was 67.5% which is superior to the result from conventional RT of which median LC duration was reported as 2 months [8, 9]. Various reports have shown that the proximity of tumor to spinal cord is related to local failure in the epidural space after SABR [11, 12]. Therefore, only tumors farther than 3-5 mm from the spinal cord were considered eligible for spinal SABR [13, 14]. However, under the consideration of salvage re-irradiation, SABR for spinal metastasis from HCC with RGCC ≥ III might be valid modality.

Although worse LC resulted from lower D_min_ at GTV in the present study implies the dose-response relationship of spinal metastasis from HCC, no optimal RT dose scheme for spinal metastasis from HCC has been determined. A few studies reported that SABR provided better LC comparing with conventional RT or even high-dose RT of 50 Gy in 10 fractions [8, 9]. However, large-scale prospective studies are necessary to confirm the optimal dose scheme.

**Figure 2:**
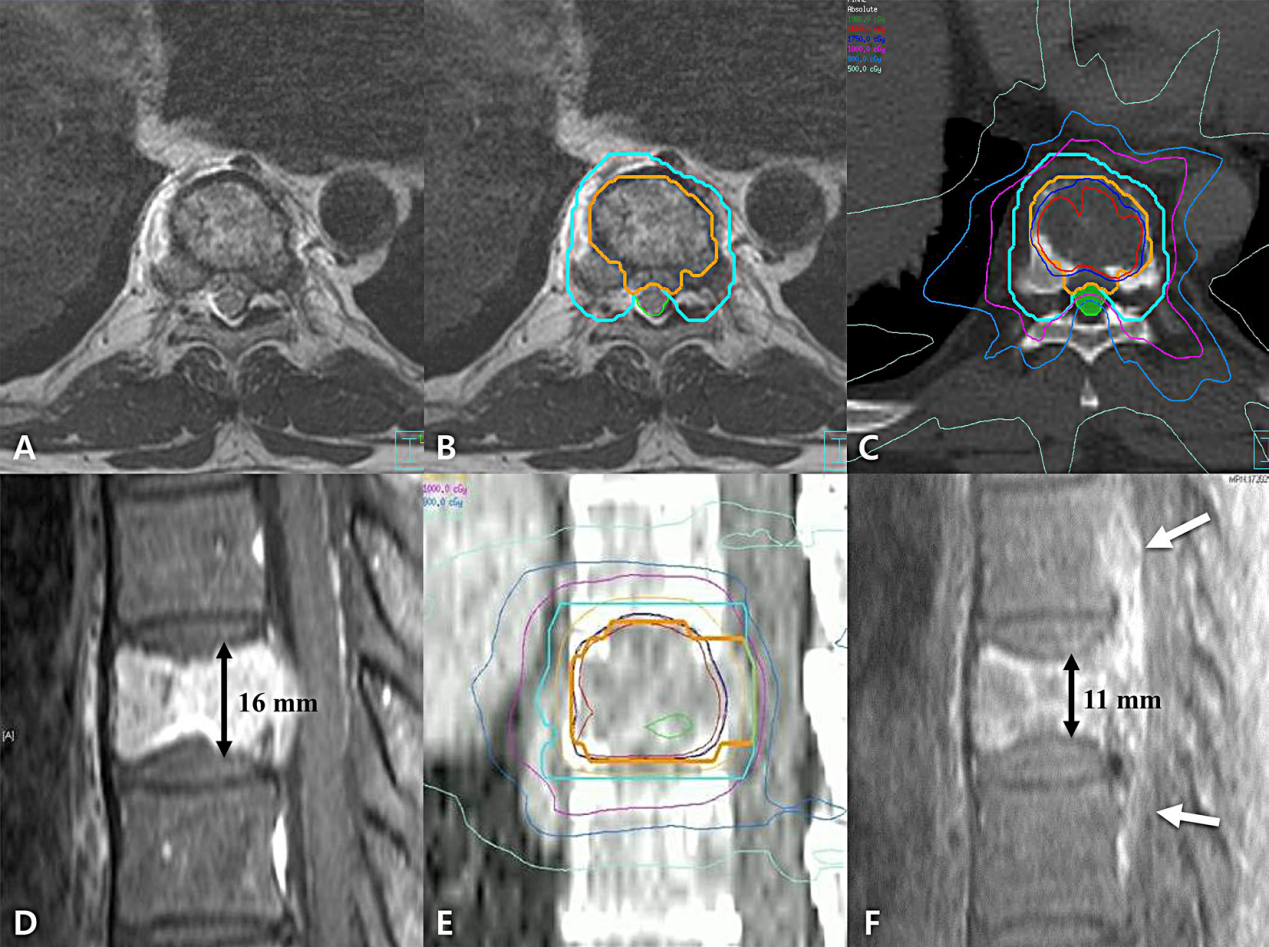
**(A)** Axial view of planning magnetic resonance image (MRI), **(B)** example of target delineation in axial view, **(C)** example of dose distribution in axial view, **(D)** sagittal view of planning MRI, **(E)** example of target delineation and dose distribution in sagittal view, **(F)** sagittal view of follow-up MRI at 3 months after stereotactic ablative body radiotherapy (SABR). The patient, who was 60-year-old male with spinal metastasis with radiographic grade of cord compression III, received SABR of 18 Gy in 1 fraction. Gross tumor volume (GTV), clinical target volume, and spinal cord was delineated in orange, sky blue, and green color, respectively. Because the GTV contacts with spinal cord, we concerned the spinal cord delineation more than GTV delineation. The GTV and spinal cord volume are exclusive to each other (B). The spinal cord dose constraint was also more concerned rather than GTV dose for spinal cord saving (C). Before SABR, we observed the tumor infiltration along the posterior longitudinal ligament (PLL) (D). The tumor progression was shown in both cranial and caudal directions along the PLL at 3 months after SABR (white arrow; F). The patient had pre-existing vertebral compression fracture (VCF) with Spinal Instability Neoplatic Score of 8 (D). There is decrease in height of vertebra body from 16 mm to 11 mm at 3 months after SABR which means the progression of pre-existing VCF (F).

Among the 6 lesions showing progression in the spinal epidural space, 3 lesions initially infiltrating along the posterior longitudinal ligament presented progression in both cranial and caudal directions without involvement of adjacent vertebral bodies (Figure 2). Various studies reported no tumor progression at the immediate adjacent vertebral level near the involved vertebra [11, 15, 16], and thereby did not recommend to include the adjacent vertebral bodies as clinical target volume (CTV) [17]. In those 3 cases, however, it is considerable to extend CTV in both cranial and caudal directions for encompassing the adjacent area along the posterior longitudinal ligament to reduce the risk of local failure.

In the present study, the 1-year VCF-free rate was 71.6%. In other studies, the VCF-free rate after SABR was reported to be 61-81% [7, 18, 19]. Few studies, however, have evaluated the risk of VCF after SABR for spinal metastasis from HCC. And also those studies have very small sample size, or reported VCF rate containing the outcome of various RT regimens other than SABR [7, 8]. To our knowledge, the present study is the first to report the risk of VCF after SABR for spinal metastases from HCC specifically. The results showed the accordance with other studies that identified pre-existing VCF and osteolytic change as significant factors of post-SABR VCF [7, 18, 19]. Considerable post-VCF rates in our study can be explained by soft-tissue formation and the osteolytic nature of spinal metastasis from HCC [4, 5, 20]. Osteolytic change makes vertebra weaker to compressive stress. And the shrinkage of metastatic soft-tissue after SABR could lead to vertebral collapse, because the soft-tissue mass itself provides resistance to the compressive stress in the involved vertebra. In the present study, 33.3% of segments with post-SABR VCF required an intervention. Therefore, early preventive vertebroplasty is considerable, especially for segments with pre-existing VCF or only-lytic changes.

In the present study, there is no observed radiation-induced neurotoxicity even in the patients who received salvage re-irradiation. This may be due to the RT planning protocol which gives priority to spinal cord dose constraint rather than GTV dose for spinal cord saving. However, whether 7 months of follow-up duration is sufficient to discuss the radiation-induced neuropathy is questionable. The latency periods of neuropathy after RT have been reported in wide range, mainly from one to several years [21]. For SABR, on the other hand, shorter latency period (0.6 year) of radiation-induced neuropathy was reported although the data is very scares [22]. Therefore, the follow-up period might be sufficient to discuss the radiation-induced neuropathy, although longer follow-up duration may be necessary to confirm the risk for radiation-induced neuropathy of SABR.

The pain response rates of spine SABR have been reported as 67-100% with various doses [14]. However, those rates were not under consideration of the confounding effect of analgesics. After adjustment of analgesic effect, pain response rates after RT with various doses were reported as only 37-50% [23]. In the present study, the pain response rate according to criteria of IBMCG was 73.3% with median pain control duration of 7 months after SABR. The median NRS also decreased after SABR significantly. Although prospective result or randomized trial is necessary, SABR might be more efficacious in pain relief and durability of pain control than RT of high-dose in various fractionation, or even 8 Gy in single fraction which showed median duration of pain control as 3 months [5, 24, 25], especially for the patients to whom higher-dose RT is recommended.

Considering that the aim of spinal SABR is long-term LC and pain-control without post-SABR VCF, it is important to select the appropriate patients expected to be long-term survivors without any morbidities. The prognosis of HCC depends on both tumor controllability and residual liver function, which are covered in the CLIP scoring system [26]. Our study identified CLIP score as an independent predictor of OS. Therefore, optimal outcome is expected to be achieved for patients with CLIP ≤ 2, the spine metastasis of RGCC ≤ II, and neither pre-existing VCF nor only-osteolytic lesion.

There are some limitations in the present study. There is an inherent bias, because the present study was a retrospective one from a single institute with a small sample size. Especially, the small number of medical records available for the analysis of pain response was a significant limitation. Another limitation is related to the systemic modality, especially sorafenib. Although our study showed no significant association between use of sorafenib and oncologic outcome, confounding effects could not be excluded.

In conclusion, SABR for spinal metastases from HCC is effective for pain relief and durable pain control. It is also efficacious in LC, especially for spinal metastases with RGCC ≤ II. Although SABR for spinal metastases with RGCC ≥ III showed poor LC, salvage re-irradiations are feasible. Because patients with normal liver function and controllable hepatic tumor status are expected to be long-term survivors, selection of appropriate patients can lead to achieve long-term survival without disease progression. As post-SABR VCF occurred frequently for vertebral segments with pre-existing VCF and only-lytic change, early intervention is considerable for those high-risk segments. SABR is a potentially valid modality for spinal metastases from HCC.

## MATERIALS AND METHODS

### Patients

Thirty-three lesions in 42 spinal segments from 29 patients were reviewed retrospectively. Patients of the study were diagnosed with spinal metastasis from HCC and received SABR for spinal metastasis between September 2009 and January 2015. Patients were diagnosed by computed tomography (CT), magnetic resonance imaging (MRI), positron emission tomography (PET)/CT, or biopsy. Initial severity of spinal cord compression was evaluated by the RGCC system proposed by S. Ryu [27, 28]. Baseline instabilities of spinal segments were evaluated according to the SINS developed by the Spine Oncology Study Group [29]. Initial pain status was assessed with NRS score. Neurological symptoms were graded by neurological grade system suggested by Ryu S et al [27, 28]. Inclusion criteria for the study group were (1) spinal metastasis with involvement of 1-3 spinal segments, (2) ambulant status, (3) Child-Pugh classification A or B, and (4) no requirement for immediate stabilization of the spine.

### Treatments

All patients underwent simulation with both CT and MRI scans sequentially with immobilization system. Slice thickness of CT scan was 2.5 mm. T1- and T2-weighted images were obtained during simulation MRI scanning, and fused with the simulation CT images.

GTV and CTV were delineated according to the Radiation Therapy Oncology Group (RTOG) 0631 protocol [13]. There was no margin expansion between CTV and planning target volume. Normal organs near the target were delineated for evaluation of dose of organs-at-risk (OARs). If the tumor contacts with spinal cord, we concerned the spinal cord delineation more than tumor volume delineation. The GTV and OAR volume are exclusive to each other.

The prescription doses were 16-20 Gy in 1-fraction, or 18-45 Gy in 3-fractions. Dose-constraints of OARs also followed the RTOG 0631 protocol. We intended to deliver a prescribed dose covering at least 90% of the GTV; if this was not possible due to spinal cord dose, we gave priority to spinal cord dose constraint rather than GTV dose. We used intensity-modulation with an inverse planning method. Every SABR was performed by Novalis Tx™ (Branilab AG, Heimstentten, Germany). For verification of setup, cone-beam CT and Exactrac were used before each treatment.

### Evaluation of treatment response and toxicity

Patients were followed with CT, MRI, or PET/CT every 1-3 months after SABR. We defined LC as neither progressive disease nor metabolic progressive disease status according to revised Response Evaluation Criteria in Solid Tumors criteria [30], or PET Response Criteria in Solid Tumors criteria [31]. Local failure was defined as progression within the 75% isodose line. LC duration was the time interval from the SABR to time of disease progression. We censored the patients who did not show local failure at the date of last visit. OS duration was defined as the interval between the SABR and time of death, or last visit if the patient was alive.

We assessed the pain response by change in NRS score at 1 week after SABR and every 1-3 months. To adjust the confounding effects of analgesics, we evaluated the pain response according to categories of the IBMCG [32]. To apply these categories, we calculated the OMED of all analgesics administrated to patients before and after the SABR.

We defined post-SABR VCF as new development of *de novo* VCF or progression of pre-existing VCF shown as shortening of vertebral body height after SABR. VCF-free duration is the time to VCF from SABR or last follow-up date if no VCF was shown. We evaluated other complications according to the Common Terminology Criteria for Adverse Events, version 4.0.1.

### Statistical analysis

Descriptive statistics were used to assess patient and tumor characteristics. LC rate, OS rate, and VCF-free rate were estimated by the Kaplan-Meier method. Univariate analysis was performed by log-rank test for potential predictors. A multivariate Cox regression model was applied to determine the independent prognostic factors associated with LC, OS, and VCF-free rate. For any 2 variables significantly correlated with each other, one of the variables was excluded from multivariate analysis. Any variables showing no event were also excluded from multivariate analysis. Mann-Whitney test was applied to evaluate the significance of the change in NRS and OMED before and after SABR. All *P*-values in this report were two-sided. Results were considered significant if *P* < 0.05. We used SPSS version 22 (IBM, Armonk, NY, USA) for all statistical analyses.

## Abbreviations

HCC: Hepatocellular Carcinoma
OS: Overall Survival
RT: Radiotherapy
SABR: Stereotactic Ablative Body Radiotherapy
VCF: Vertebral Compression Fracture
LC: Local Control
CLIP: Cancer of the Liver Italian Program
RGCC: Radiographic Grade of Cord Compression
D_min_: Minimal Dose
GTV: Gross Tumor Volume
Gy_10_: Biologically Effective Dose with α/β of 10
SINS: Spinal Instability Neoplastic Score
NRS: Numeric Rating System
OMED: Oral Morphine Equivalent Dose
IBMCG: International Bone Metastases Consensus Group
CTV: Clinical Target Volume
CT: Computed Tomography
MRI: Magnetic Resonance Imaging
PET: Positron Emission Tomography
RTOG: Radiation Therapy Oncology Group
OAR: Organ at Risk
ECOG: Eastern Cooperative Oncology Group
BM: Bone Metastasis
VCFFR: Vertebral Compression Fracture-Free Rate
HR: Hazard Ratio
CI: 95% Confidence Interval
NA: Not Analyzed
